# Thrombolysis in an Acute Ischemic Stroke Patient on Direct Anticoagulant Therapy Outside of the Traditional Time Window: A Case Report

**DOI:** 10.7759/cureus.29673

**Published:** 2022-09-27

**Authors:** Sofie Moorthamers, Nancy Mattar, Laeticia Frezals, Thierry Preseau, Marie-Dominique Gazagnes

**Affiliations:** 1 Emergency Department, Brugmann University Hospital, Brussels, BEL; 2 Emergency Department, Erasmus Hospital, Brussels, BEL; 3 Emergency Medicine, Lebanese University Faculty of Medicine, Okaibe, LBN; 4 Neurovascular-Stroke Department, Brugmann University Hospital, Brussels, BEL

**Keywords:** time-to-treatment, neuroimaging and neurointervention, oral anticoagulation, mild acute ischemic stroke (ais), systemic thrombolysis

## Abstract

A stroke is a life-threatening medical condition that could be disabling if left untreated. Intravenous thrombolysis (IVT) and mechanical thrombectomy (MT) can be effective when initiated in an acute stroke, but their benefit is time-dependent and their use may be restricted by contraindications (CIs) such as anticoagulation therapy. The critical therapeutic time window, which was previously limited to 4.5-6 hours, is now extended to 24 hours in selected patients due to the development of advanced neuroimaging techniques. Herein, we discuss the case of a 50-year-old patient on rivaroxaban who developed acute ischemic stroke (AIS) and was treated successfully with intravenous recombinant tissue plasminogen activator thrombolysis more than six hours after the time he was last seen well (LSW). Our case demonstrates the importance of advanced neuroimaging techniques in identifying AIS candidates for IVT and/or MT with late or unknown time windows as well as the importance of case-by-case assessment when challenged by theoretical contraindications for thrombolysis.

## Introduction

Worldwide, stroke is the second leading cause of death and the third leading cause of disability [1]. Almost 80% of all strokes are ischemic in etiology. Intravenous thrombolysis (IVT) and mechanical thrombectomy (MT) have shown effectiveness in the treatment of acute ischemic stroke (AIS). Their benefit, however, is time-dependent and any delay in diagnosis and treatment can have devastating effects on AIS outcome. Therefore, early recognition and treatment initiation are the keys to reducing morbidity and mortality associated with AIS [2]. Until recently, the time to initiate IVT was limited within 4.5 hours after stroke onset. However, in selected patients and based on advanced neuroimaging criteria, time windows used for thrombolytic therapy and thrombectomy are now being extended [3,4]. Every sixth stroke is the consequence of atrial fibrillation (AF). Although direct oral anticoagulants (DOAC) are highly effective for stroke prevention in patients with nonvalvular AF, 1-2% of patients on DOAC will have AIS [5]. AIS in DOAC-treated patients is rare, but when it occurs, choosing the most suitable treatment represents a challenge for clinical physicians. Currently, there is only a little evidence to guide clinicians in decision-making regarding IVT or MT in AIS patients on DOAC. Moreover, anticoagulant therapy, if the dose was received less than 48 hours, is theoretically a contraindication (CI) for IVT since patients on anticoagulants were excluded from all randomized controlled trials studying IVT. Nonetheless, there is slowly growing evidence that IVT is feasible and safe with a good clinical outcome in AIS patients on DOAC [6].

## Case presentation

**Figure 1:**
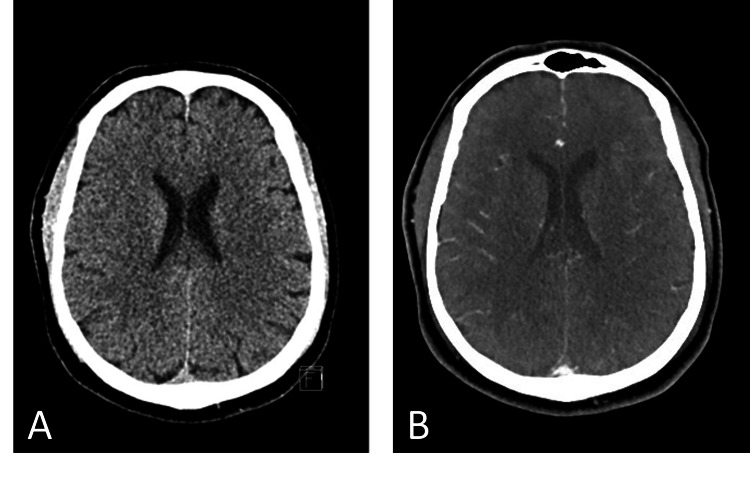
NCCT (A) and CTA (B) images showing no signs of intracranial hemorrhage, brain ischemia or artery occlusion. CTA: CT angiography; NCCT: conventional brain non-contrast computed tomography.

At 5:15 pm, a 50-year-old man was brought to the emergency department (ED) for a suspected stroke. The initial patient assessment revealed a blood pressure of 142/81 mm Hg, a heart rate of 110 beats per minute, oxygen saturation of 100% on room air, a body temperature of 36.2 °C, and a fingerstick glucose level of 132 mg/dL. The patient was conscious, alert, and oriented times three, with a Glasgow Coma Scale of 4-2-6 (12), pupils equal and reactive to light, left lower facial palsy, severe dysarthria, left hemiparesis, anosognosia, profound hemi-attention towards the left, and a National Institutes of Health Stroke Scale (NIHSS) score of 12. The latter score was calculated according to the following clinical findings: partial paralysis of lower face: +2, some effort against gravity left arm: +2, no effort against gravity left leg: +3, severe dysarthria: +2, LOC questions: +1, and profound hemi-inattention: +2. He was on 20 mg of rivaroxaban once daily for non-valvular paroxysmal AF and the last dose was taken at 8:00 pm the day before. History went back to 4:27 pm when the patient was found unable to respond appropriately after a post-lunch nap. The last time he was seen well (LSW) was around 12:00 pm and the presumed onset of stroke was between 12 and 24 hours after the last dose of rivaroxaban. The estimated pre-stroke modified Rankin Scale (mRS) score was 0. Conventional brain non-contrast computed tomography (NCCT) performed at 5:22 pm was normal, i.e., without signs of intracranial hemorrhage (ICH) or brain ischemia (Figure 1A), and CT angiography (CTA) revealed no large artery occlusion (LAO) (Figure 1B).

**Figure 2:**
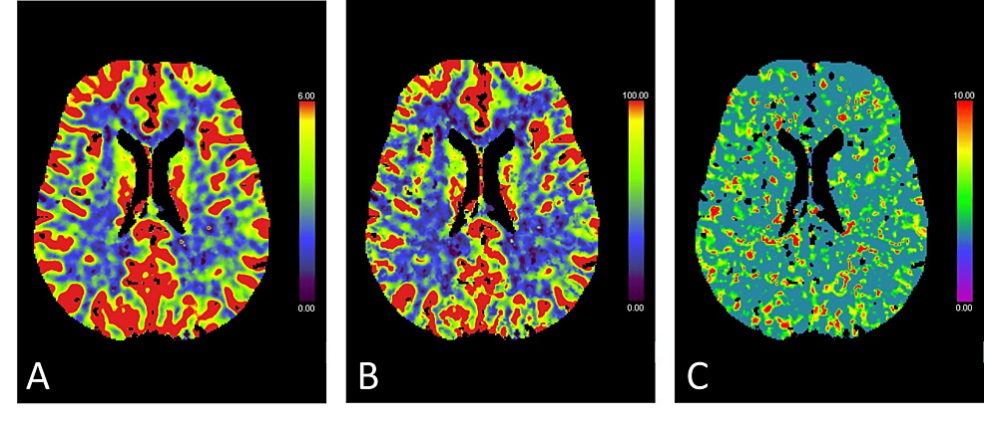
CT perfusion images (A: cerebral blood flow, B: cerebral blood volume, C: mean transit time) revealing no brain ischemia.

Subsequent CT perfusion (CTP) imaging showed no perfusion deficit (Figure 2).

**Figure 3:**
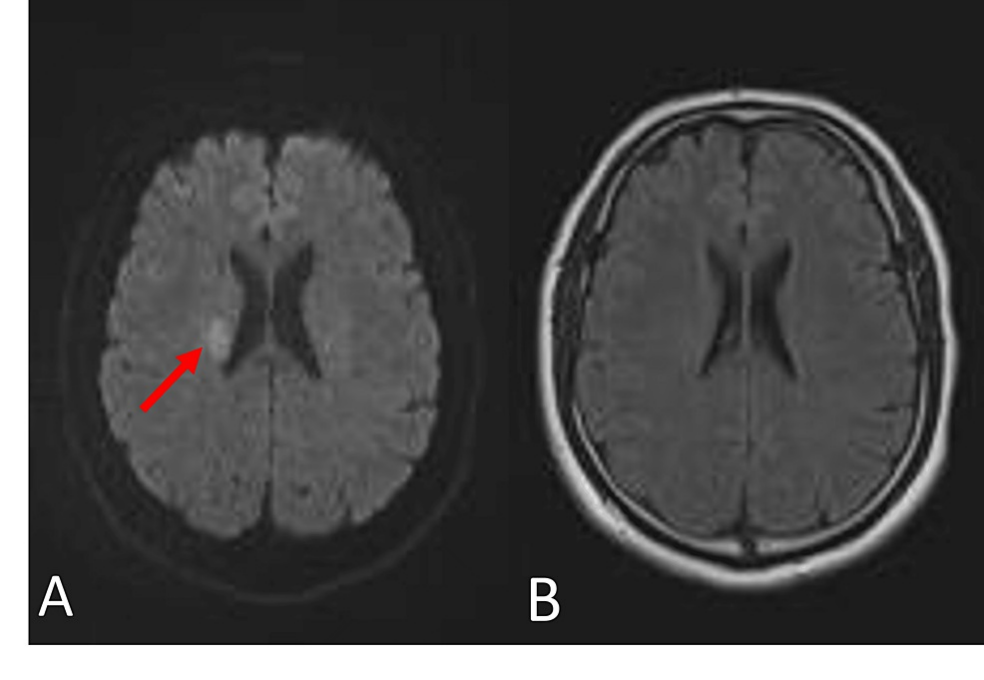
MRI of the brain showing “mismatch” between (A) axial DWI demonstrating a small focus of high signal intensity in the right internal capsule and putamen (red arrow) and (B) FLAIR image with no visible signal change.

Laboratory tests revealed an international normalized ratio (INR) of 0.96, prothrombin time (PT) of 10.2 seconds (control: 9.9-11.8 seconds), activated partial thromboplastin time (aPTT) of 22.9 seconds (normal: 20.3-28.3 seconds), platelet count of 241 × 103 mm^3^ (normal: 150 - 440 × 10^3^ mm^3^), and creatinine level at 0.86 mg/dL with an estimated glomerular filtration rate of >90 mL/min/1.73 m^2^. Anti-Xa activity and rivaroxaban concentration were 0.09 U anti-Xa/mL and < 20 ng/mL, respectively. Electrocardiography showed regular sinus rhythm. The possibility of urgent brain magnetic resonance imaging (MRI) was discussed and, at 06:39 pm, brain MRI revealed an MRI "mismatch" between diffusion-weighted MRI (DW-MRI) and fluid-attenuation inversion-recovery (FLAIR) acquisitions in the territory of the right middle cerebral artery (MCA). DW-MRI demonstrated a small focus of high signal intensity in the right internal capsule and putamen, with no visible signal change on FLAIR images (Figure 3).

**Figure 4:**
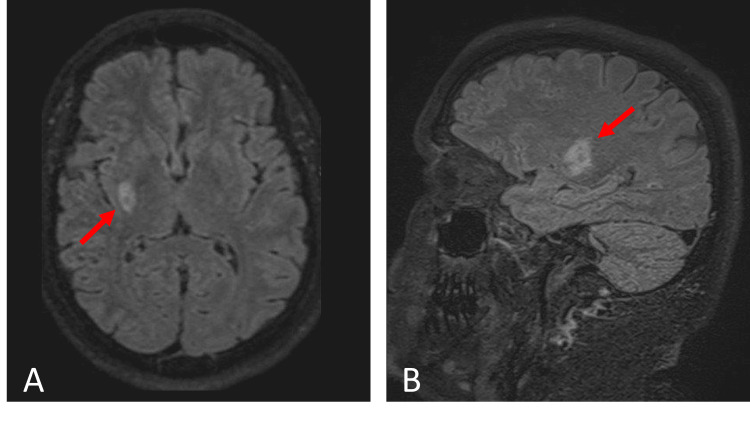
MRI one month later revealing a FLAIR hyperintensity (red arrows) consistent with right striatocapsular infarction.

At 06:43 pm, the neuroradiologist confirmed the presence of an acute lacunar ischemic stroke in the right internal capsule and putamen. The findings were discussed with the stroke specialist, and the patient was deemed a good candidate for IVT. At 06:49 pm, with the patient’s informed consent, an IV dose of 0.9 mg/kg rt-PA, with 10% as a bolus and the rest as an infusion over one hour, was given. This treatment was administered 22 hours after the last dose of rivaroxaban and more than six hours after LSW. During IVT, improvement in the patient's neurological deficit was observed. At 07:40 pm, complete neurological recovery (NIHSS score of 0) occurred. The patient was then hospitalized at the primary stroke unit for tension control. A brain CT performed 24 hours post-IVT revealed no hemorrhagic change. Six days later, the patient was discharged home from the hospital with NIHSS and mRS scores of 0 and without neurological deficit. For secondary stroke prevention, anticoagulant therapy was switched to dabigatran because of the availability of its specific antidote and due to previous treatment failure of rivaroxaban. A brain MRI one month later revealed a FLAIR hyperintensity consistent with right striatocapsular infarction in the patient who was asymptomatic due to the performed IVT (Figure 4).

## Discussion

The development of DOACs with their antidotes and the increased access to advanced neuroimaging techniques has led to an ongoing process of reconsideration of eligibility for IVT and/or MT in AIS. Appropriate appraisal of absolute and/or relative CIs to IVT and/or MT has become decisive in acute stroke care since "yes/no" to treatment decisions can have a profound impact on the outcome of AIS patients.

Neuroimaging

For all patients with suspected stroke, brain imaging evaluation to exclude intracranial hemorrhage (ICH) or other abnormalities should be performed as quickly as possible and before any recanalization therapy [2,7]. In patients who may be eligible for IVT and who arrive within 4.5 hours of LSW or stroke symptom onset, brain NCCT is effective in excluding ICH and it is sufficient as a neuroimaging modality for decisions about IVT initiation. For these patients, additional multimodal neuroimaging, such as CTP and MRI perfusion imaging, is not necessary and may delay the time-sensitive initiation of IVT [2,7]. For patients with an unknown onset of stroke (UOS), including wake-up stroke and unwitnessed daytime stroke, more than 4.5 hours from LSW, MRI "mismatch" or DWI-positive FLAIR-negative lesions can identify those patients who may benefit from IVT within 4.5 hours of stroke symptom recognition [3]. In some UOS patients with a favorable perfusion-imaging profile on CTP or perfusion-diffusion MRI, the treatment window for IVT can even be extended to nine hours after the onset of stroke or on awakening with stroke (if awakening within nine hours from the midpoint of sleep) [4]. During initial imaging evaluation and before obtaining serum creatinine concentration, non-invasive head and neck vessel imaging is recommended for patients who may be eligible for MT. CTA may be performed during IVT and should not be delayed [2,7]. For patients who may be eligible for MT and arrive between 6 and 24 hours after LSW, CTA combined with CTP or MRA with DW-MRI with or without MR perfusion is recommended for selecting MT candidates according to DWI or CTP Assessment with Clinical Mismatch in the Triage of Wake-Up and Late Presenting Strokes Undergoing Neurointervention with Trevo (DAWN) or Endovascular Therapy Following Imaging Evaluation for Ischemic Stroke 3 (DEFUSE 3) eligibility criteria [2,7]. In our case, on ED arrival, the patient was transported directly to the CT examination room for NCCT. Door-to-image time was seven minutes. Neither ICH nor stroke mimics were detected. Thus, CTA was performed to look for LAO and eligibility for MT within the six hours of the LSW treatment window. However, CTA showed no LAO, and thus MT as a treatment strategy was ruled out. Since IVT could be a salvage treatment option in this patient if DOAC concentration was sufficiently low, CTP was performed but no perfusion deficit could be demonstrated. However, when laboratory test results were available and the rivaroxaban concentration turned out to be less than 20 ng/mL, brain MRI was still indicated to evaluate IVT eligibility in this patient. The time of LSW was almost six hours and CTP was negative for stroke, but, contrary to MRI and due to resolution issues, deep and/or small lacunar infarctions may be poorly visualized on CTP [8]. Moreover, MRI is more sensitive than CTP to the early parenchymal changes of brain ischemia [9]. So, at 06:39 pm, an emergency brain MRI was performed, revealing an MRI "mismatch" in the right internal capsule and putamen.

IVT eligibility

The benefit of IVT and/or MT is time-dependent. Thus, treatment should be initiated as quickly as possible and the time to initiate IVT and/or MT is limited. Recently, the American Heart Association and American Stroke Association (AHA/ASA) guidelines recommend, in patients with DAWN or DEFUSE 3 trial inclusion criteria, MT beyond the classical six-hour time window, up to 24 hours after LSW. For IVT, intravenous recombinant tissue plasminogen activator (rt-PA) initiation within 4.5 hours of LSW remains the standard of care for most AIS patients [2]. Despite the verified benefits of IVT, globally only 8-10% of all AIS patients receive IVT [10]. This is largely due to delays in presentation time >4.5 hours from LSW [10,11]. The AHA/ASA restrictive time window for IVT (and most other IVT exclusion criteria) is derived from the National Institute of Neurological Disorders and Stroke (NINDS) trial in 1995 and the European Cooperative Acute Stroke Study 3 (ECASS III) trial in 2008. However, in order to increase IVT treatment rates in AIS, neuroimaging approaches to identify IVT-eligible patients beyond the 4.5-hour treatment window have been developed. In 2018, following the Efficacy and Safety of MRI-based Thrombolysis in Wake-up Stroke (WAKE-UP) trial, the AHA/ASA extended the IVT time window to 4.5 hours from stroke symptom recognition for patients with DW-MRI lesions smaller than one-third of the MCA territory and with no signal change on FLAIR [2,3]. More recently, the 2019 Extending the Time for Thrombolysis in Emergency Neurological Deficits (EXTEND) trial even suggests that IVT may be considered for patients with AIS between 4.5 and 9 hours from LSW and with a "penumbral mismatch" identified by MRI or CTP [4]. As illustrated in this case, in selected patients, IVT can be safe outside the classical 4.5-hour time window from stroke onset. It equally shows the need to constantly review the absolute and relative CIs to IVT, many of which are not based on evidence but rather derived from expert opinion and exclusion criteria for major stroke trials, such as the NINDS trial dating from 1995, which led the FDA to approve rt-PA within three hours of stroke [6,11,12]. Observational studies and clinical practice over the past 25 years have led to mounting evidence that several current CIs and limitations for IVT are overly restrictive and that IVT can be performed safely in patients previously deemed ineligible for IVT [11,12]. Moreover, IVT CIs may vary among AHA/ASA guidelines, European Stroke Organization (ESO) guidelines, and rt-PA insert [2,7,11,12]. Historically, anticoagulant treatment in AIS patients presents a formal CI for IVT. Patients on a full treatment dose of low-molecular-weight heparin (LMWH) within the previous 24 hours should not receive IVT [2]. For AIS patients on vitamin K antagonists (VKA), however, IVT may be reasonable with an acceptable benefit-risk ratio up to an INR of 1.7 [2]. For patients on DOAC, knowledge about the safety of IVT is limited since no large cohorts exist and all major IVT trials were conducted in the era before any DOAC became available [6,13]. Nevertheless, it is important to establish an approach to guide the use of IVT in AIS patients on DOAC since a relevant portion of these patients may have only low or absent anticoagulant activity and could benefit from IVT without an elevated bleeding risk [6,14]. Current international guidelines warn against IVT administration in patients on DOAC unless sensitive laboratory tests are normal or the patient (with normal renal function) has not received DOACs for more than 48 hours before the stroke [2]. Thus, for patients with a normal renal function who stopped taking the DOAC for at least 48 hours, stroke management should not differ from that of patients without DOAC treatment [2,15]. DOACs have great pharmacodynamic and pharmacokinetic predictability, a rapid onset of action (with peak levels < 4 hours after intake), and similar short half-lives (5-17 hours) [16]. Thus, when a patient’s last dose of DOAC is more than four half-lives ago, a very low probability of clinically relevant DOAC plasma level is expected and IVT can be safely performed [2,6,16]. Conversely, IVT with <48 hours of DOAC intake is regarded as off-label. But IVT might be considered for AIS with recent DOAC intake or with impaired renal function if sensitive specific DOAC anticoagulation tests indicate low or absent antithrombotic activity [2]. Routine coagulation tests, including PT, aPTT, and TT, cannot be used to quantify the anticoagulant effects of DOACs. These commonly used coagulation assays lack the necessary sensitivity and specificity to reliably measure DOAC anticoagulant activity; however, they can be used, in the absence of specific tests, to qualitatively rule out any residual drug effect [14,16,17]. In order to effectively quantify the anticoagulant effect of DOACs, plasma drug concentrations, ecarin clotting time, dilute thrombin time, and/or DOAC calibrated chromogenic anti-FXa assays are recommended [2,16,17]. Although the determination of DOAC plasma levels is now feasible, generally accepted thresholds that allow safe IVT in AIS patients on DOAC still need to be validated. An early recommendation in 2013 proposed plasma concentrations of 10 (apixaban), 50 (dabigatran) and 100 (rivaroxaban) ng/mL as cut-offs for considering IVT [18]. In 2017, a study on rivaroxaban patients stated that IVT should be recommended, considered, or avoided if rivaroxaban plasma levels were <20, 20-100, and >100 ng/mL, respectively [19]. A more recent practical guideline from the European Heart and Rhythm Association in 2018 suggests IVT in selected patients on rivaroxaban, apixaban, or edoxaban if plasma levels measured more than four hours after the last drug administration are less than 30 ng/mL [20]. In this case, the rivaroxaban level was <20 ng/mL, and the patient was considered eligible for IVT.

## Conclusions

The benefit of IVT and/or MT in AIS is time-dependent, and the clock for administering these time-sensitive therapies begins with the time of LSW. With the development of advanced neuroimaging techniques, the critical time windows of 4.5 hours and 6 hours for IVT and MT, respectively, can be extended up to 24 hours in selected patients that adhere to EXTEND, WAKE-UP, DAWN, or DEFUSE 3 trial neuroimaging eligibility criteria. To identify AIS candidates for IVT and/or MT in late and unknown time windows, the emergency physician (EP) should be familiar with advanced neuroimaging techniques and should know their specific relevance, their indications, and their different limitations. Similarly, the EP should be aware that exclusion criteria for IVT vary widely and that several historical CIs have already undergone a critical reappraisal with subsequent scope extension for IVT. Currently, with the development of DOACs and their antidotes, the need to reconsider the eligibility of AIS patients on DOACs has become a highly pertinent topic. Although current guidelines warn against IVT in patients treated with DOAC, IVT can be safe in selected AIS patients on DOAC, as illustrated in this case. However, further clinical trials are warranted to better delineate the contraindications of IVT and/or MT in patients on DOAC.
